# Health Inequities in Pediatric Trauma

**DOI:** 10.3390/children10020343

**Published:** 2023-02-09

**Authors:** Mark L. Kayton, Victoriya Staab, Brandon Stahl, Khea Tan, Larissa Russo, Meagan Verney, Margaret McGuire, Harpreet Pall

**Affiliations:** 1 K. Hovnanian Children’s Hospital, Jersey Shore University Medical Center, Neptune, NJ 07753, USA; 2 Departments of Surgery and Pediatrics, Hackensack Meridian School of Medicine, Nutley, NJ 07110, USA

**Keywords:** health equity, pediatric trauma care, access to care, gun violence, child abuse, head trauma, burn injuries

## Abstract

This review article highlights the disparities evident in pediatric trauma care in the United States. Social determinants of health play a significant role in key aspects of trauma care including access to care, gun violence, child abuse, head trauma, burn injuries, and orthopedic trauma. We review the recent literature as it relates to these topics. The findings from these recent studies emphasize the important principle that trauma care for children should be designed with a focus on equity for all children.

## 1. Introduction

Social determinants of health (SDOH) encompass five key domains: economic, education, social and community context, health and health care, and built environment. There is a recognition that health starts in our homes and communities and is further shaped by a myriad of factors. Social factors have been proven to significantly influence health across various populations and settings, in addition to medical care [1]. This has led to an explosion of research attempting to understand the interactions between medical interventions, outcomes, and SDOH. Previous research has brought existing health care disparities to the forefront of national discussions and emphasized that health equity needs to be a priority in future research and design of health care delivery. Health equity can be measured by evaluating the prevalence of health disparities [2]. When health disparities are identified and reduced, health is improved in economically and socially disadvantaged individuals, and progress is made toward achieving health equity [2].

This review article emphasizes the existing SDOH and disparities seen in pediatric trauma care. Identifying social determinants that influence health disparities in pediatric trauma can help reveal barriers to care [3]. If barriers to care are identified, appropriate measures can then be taken to potentially improve care and outcomes in underserved groups, which in turn helps achieve progress toward health equity [3]. We review recent literature specifically as it relates to access to care, gun violence, child abuse, head trauma, burn injuries, and orthopedic trauma in the United States. These represent important topics in pediatric trauma relating to medical distribution, injury mechanism, and injury site. An abundance of previous research has been published examining existing disparities within these areas.

## 2. Access to Care

It is well understood now that specialized care, equipment, and personnel are needed to optimize care for the pediatric population. This awareness has led to the growth in pediatric trauma centers (PTC) over the past few decades. Children and adolescents (age <18 years) account for approximately 22% of the United States’ population and overwhelmingly the leading causes of death are injury-related mechanisms [4,5]. In 2016, over 60% of deaths among children under the age of 15 were from injury-related causes, including motor vehicle crashes and firearm-related injuries [5]. Since then, this number has only increased, especially with the rapid uptick in firearm-related deaths since the start of the COVID-19 pandemic [6,7]. Despite this, there continues to be a shortage of PTCs relative to the number of adult trauma centers (ATC). Currently the American College of Surgeons (ACS) has verified 37 PTCs across the United States; however, there are additional centers that do not follow the ACS verification system and those that self-classify as PTCs for a total of 170 unique PTCs across 41 states. The lack of accessible centers portends to worse outcomes when evaluating the benefit of a PTC versus an ATC. Mortality rates in pediatric injury were found to be inversely correlated with a state’s number of verified PTCs [8]. Potoka et al. [9] evaluated 13,351 injured children who were treated at a PTC or an ATC with added qualifications to treat children (ATC AQ) and found overall survival was significantly better at PTC and ATC AQ when compared to ATC (Level 1 and 2). The mortality rate for Injury Severity Score (ISS) > 15 was significantly higher in ATC I (21.6%) and II (16.2%) compared to PTC (11.9%) and ATC AQ (12.4%). When stratified by age, children treated at a PTC after blunt trauma between 10–14 years of age experienced significantly lower mortality rates (6.7%) in comparison to all other trauma centers, including ATC AQ (12.5%, ATC I 16.2%, ATC II 12.5%). Given the similar mortality rates seen in PTC and ATC AQ, Oyetunji et al. [10] aimed to evaluate treatment outcomes of children treated at ATC AQ compared to ATC and found that there was a 20% risk reduction in mortality when children were treated at an ATC AQ, as well as improved survival in children with severe traumatic brain injuries (Glasgow coma score <8). Within the adolescent population (15–18 years of age), these patients were found to have less surgery and lower rates of mortality if cared for at a PTC when compared to an ATC, with no differences in complication rates or length of stay longer than 4 days [11]. Furthermore, children with severe head, splenic, or liver injuries were found to have better outcomes when treated at a PTC compared to an ATC [9,12]. Therefore, despite the low number of PTCs relative to ATCs, studies suggest that outcomes are improved when children are evaluated and treated at PTC/ATC AQs. Thus, the need for appropriate access to these specialized centers in a timely fashion in order to optimize care continues to rise.

When plotted on a map of the United States, these pediatric centers are located in regions with high population densities and correlate with availability and access to PTCs and pediatric emergency departments. Rural communities are without access to a PTC or hospital with strong pediatric capabilities, leading to increased risk of poorer outcomes [13]. When stratified, children in urban environments (more population-dense) had a greater than 4-fold rate of access to PTCs than those in rural communities (93.5% compared to 22.9%, respectively). This distribution is similarly seen within the adult trauma population [13,14]. This divide in access to health care is further highlighted when considering more vulnerable populations such as Black or Hispanic patients. Historically, these groups of people are within lower socioeconomic status groups, more likely to live in poverty, and are less likely to have health insurance and thus are disproportionately affected by trauma center closures [15,16]. Hanchate et al. [15] evaluated whether racial/ethnic minorities are transported to the same emergency departments as white residents within the same zip code and found that Black and Hispanic patients were more likely to be transported to a safety-net hospital, different from that of the hospital that most white patients were transported to.

Not only does the distance to a PTC affect access, but the availability of transport also plays a role in influencing access and barriers to care in pediatric trauma. Timely transport to a designated trauma center is necessary to ensure that trauma patients can receive the appropriate level of care [17,18]. A recent study evaluating timely access to pediatric trauma care found that disparities exist in access to PTCs by ground and air transport [18]. Burdick et al. [18] determined that in 2020, 45.5% of US children less than 15 years of age (about 30 million children) could not access a level 1–3 PTC within 60 min by ground transport, while 25.9% (greater than 15 million children) could not access a level 1–3 PTC within 60 min by air transport. In addition to geographic disparities, the study identified differences in access when stratifying by race and ethnicity [18]. Overall, 89.5% of Asian children, 78% of Black children, 76.9% of Hispanic children, 71.3% of White children, 61% of Pacific Islander children, and 43.5% of American Indian/Alaskan Native children had timely access by air transport to a pediatric trauma center [18]. Further, 76.5% of Asian children, 64.2% of Black children, 60.2% of Hispanic children, 59.3% of Pacific Islander children, 48.7% of White children, and 31% of American Indian/Alaskan Native children had timely access by ground transport to a pediatric trauma center [18]. Disparities also exist between transport of pediatric trauma patients in urban and rural areas. A study by McCowan et al. [19] examining helicopter emergency medical transport (HEMS) of pediatric blunt trauma victims less than 17 years of age to three level 1 trauma facilities between 1997 and 2001 found differences in transport times between urban and rural areas. Pediatric patients transported from rural areas had longer flight times, longer scene times, and more total mileage than patients transported from urban areas [19]. A study comparing helicopter emergency medical services (HEMS) and ground emergency medical services (GEMS) in children less than 18 years of age from two level 1 pediatric trauma centers in 2003–2013 found differences in transport between children living in urban and rural areas as well [20]. The study found that rural counties utilized HEMS transport more and had to travel longer distances than urban counties [20].

Transport is also impacted by insurance coverage. Patel et al. [21] utilized data from the 2017–2018 Trauma Quality Programs Participant Use File (TQP PUF) to examine how insurance coverage (publicly or privately insured) and mode of transport (emergency medical services (EMS) vs. non-emergency medical services) are associated in pediatric trauma patients less than 18 years of age. The study matched patients based on injury, facility, and patient characteristics, and found that in comparison to insured patients, uninsured patients were more likely to utilize EMS transport but were less likely to be admitted [21]. Patel et al. [21] explained that these findings could be related to lack of availability of private transport for uninsured patients [22,23], resulting in uninsured patients being more likely to utilize EMS transport, regardless of severity of injury. Insurance coverage also plays a role in transfer of pediatric trauma patients between facilities. Hamilton et al. [24] conducted a study assessing the impact of insurance coverage on the likelihood that pediatric patients would be transferred to a level 1/2 pediatric trauma center after initially being evaluated at a lower-level facility. The study analyzed data from pediatric trauma patients less than 16 years of age in the 2007–2012 National Trauma Data Bank (NTDB) and found that after accounting for clinical and injury factors, children who lacked insurance had an increased likelihood of transfer to a major trauma facility, suggesting a potential bias in triage [24]. Another topic related to insurance status in pediatric trauma patients is inpatient rehabilitation. Inpatient rehabilitation is a crucial part of pediatric trauma care [25]. Nguyen et al. [25] examined the use of inpatient rehabilitation in pediatric trauma patients aged 0–18 admitted to University of California, San Francisco (UCSF) Benioff Children’s Hospital in Oakland with an ISS > 9 from 2004–2014. Insurance authorization was required in order to be admitted to the facility’s inpatient rehabilitation, and patients were enrolled in public insurance programs prior to admission if it was determined that they were uninsured [25]. The study found that privately insured children were more likely to receive inpatient rehabilitation services than publicly insured children [25]. In addition to impacting access to pediatric trauma care, socioeconomic factors affect access to injury prevention strategies and education. Individuals who lack the financial resources or the educational background to comprehend principles of injury prevention are less likely to buy safety equipment, carry out injury prevention practices, or think that prevention measures are necessary, resulting in increased injury risk [26]. In order to establish appropriate injury prevention programs and policies for pediatric trauma, factors affecting barriers to injury prevention in high-risk groups need to be considered.

## 3. Child Abuse

In the United States, non-accidental trauma (NAT) is fatal in more than 2 out of 100,000 children annually and can be impacted by the disparities and inequity present in healthcare [27]. Differences amongst regions, socioeconomic status (SES), insurance status, individual and community-level, SDOH, and access to care at designated children’s hospitals can influence individual outcomes in NAT. In 2018, Hymel et al. [28] conducted a cross-sectional analysis across 18 sites of the Pediatric Brain Injury Research Network (PediBIRN) that aimed to evaluate and characterize racial/ethnic disparities in the evaluation and reporting of suspected abusive head trauma (AHT). Across the 18 sites, significant racial and ethnic disparities among the evaluation and reporting practices of AHT were noted at two sites, located in different geographic areas. These differences were almost exclusive to patients presenting with “low risk” for AHT. Although the data did not confirm physician implicit bias at these sites, they were thought to reflect and support a potential case of ascertainment bias; more thoroughly evaluating suspected cases of abuse in minority race/ethnicity patients to avoid fewer missed cases [28]. This implicit bias is suggesting a racial difference in the prevalence of NAT/ AHT among patients can have detrimental sequelae such as missed NAT/AHT, prevention of earlier recognition of NAT/AHT, as well as unnecessary costs and prolonged stays in patients with non-AHT [28,29,30,31,32]. Age is also a risk factor for child abuse. A study examining the burden of non-accidental trauma on the pediatric population found that increasing age was associated with a reduced risk of mortality in children admitted to the hospital due to NAT [27]. The study obtained data on patients less than 18 years of age between 2000 and 2012 from the Kids’ Inpatient Database (KID). Another study utilizing data from the Kids’ Inpatient Database (KID) for the years 2006, 2009 and 2012 found that children 2 years of age or less were more likely than older children to be admitted as a result of NAT [33]. Secondary analysis of Medicaid data from four states has revealed that 30.6% of children with maltreatment diagnoses had been previously diagnosed with an injury. Additionally, 88.4% of infants diagnosed with maltreatment had one or more well-child visits before their maltreatment diagnosis, suggesting an opportunity for improved screening at these visits [34]. In 2022, Rebbe et al. [35] used Child Protective Services (CPS) data from Washington state ranging from 1999–2013 to evaluate the role of race/ethnicity in the reporting of child abuse to CPS and diagnostic coding of child abuse during maltreatment hospitalization. Their analysis demonstrated that children with Asian/Pacific Islander mothers were less likely to be reported to CPS than children with white mothers, with no differences between other races/ethnicities. Children with public insurance were found to have a significantly higher relative risk of being reported to CPS for child abuse when compared to children with private insurance [35]. The relationship between insurance type and child abuse had also been noted in a cross-sectional study by Henry et al. [36], in which they found that ED encounters for children diagnosed with physical abuse who had private insurance or were self-pay had a higher adjusted discharge percentage when compared to children with public insurance. Along with insurance type and status, other SDOH such as poverty, parental educational attainment, housing instability, and food insecurity have been shown to be associated with child abuse [37,38,39]. The first step in eliminating these disparities is understanding their presence and their impact on injured children, which will promote proper early detection and foster healthcare system changes that can help in the recognition and prevention of child abuse.

## 4. Gun Violence

Since 2017, firearms have been the leading cause of death in the United States among children and youth between the ages of 0–24 [40]. According to the CDC, from 2000 to 2020, firearm-related deaths among children and young adults increased from 7.30 per 100,000 to 10.28 per 100,000 [41,42]. With firearms present in 18–64% of US households varying regionally and firearm-related injuries being the leading cause of unintentional death among children, effective intervention to address this gun violence epidemic is imperative [43,44]. Fowler et al. [45] obtained data from the CDC National Vital Statistics System and National Electronic Injury Surveillance System (NEISS) to examine firearm injuries in children aged 0–17 between 2002 and 2014 and determined that differences exist in the risk of fatal firearm injury and the factors surrounding firearm homicide between younger children (0–12 years of age) and older children (13–17 years of age). The study found that older children had a higher rate of fatal firearm injury than younger children and were more likely to be the victim of a firearm homicide due to crime and violence [45]. Firearm homicide in younger children was more likely to involve intimate partner or family conflict and occur in incidents with multiple victims [45]. In 2019, Bayouth et al. [46] conducted a retrospective review of pediatric gunshot wound (GSW) data from 1996–2016 at a level 1 trauma center in order to identify risk factors for pediatric gun violence. Through mapping and cluster analysis of their data, Bayouth et al. [46] were able to conclude that impoverished and lower SES neighborhoods had higher incidences of pediatric GSW. Distressed community indices (DCI) calculated from US Census data have also been utilized to identify at-risk communities for pediatric gun violence [47]. Tracy et al. [47] identified that community-level SDOH such as the proportion of adults with high school diplomas, poverty rate, median income, and housing vacancy were highly predictive of areas having a higher incidence of pediatric gun violence. On an individual SDOH level, food insecurity and food deserts in urban regions have been associated with a higher incidence and prevalence of interpersonal gun violence [48]. However, food deserts in rural areas have not been shown to impact pediatric gun violence [48]. In 2020, Urrechaga et al. [49] conducted a retrospective review of pediatric GSW data at a level 1 trauma center in Miami, Florida from September 2013 to January 2019 and found that geodemographic analysis could be utilized to identify neighborhoods at an increased risk for pediatric gun violence. Their analysis identified these “hot spots” predominantly in underserved African American and Hispanic communities throughout Miami [49]. Despite gun violence and pediatric GSW injuries being viewed as a problem among urban teens, Choi et al. [50] conducted a retrospective review of pediatric gun violence data from a level 1 trauma center that served both a rural and urban community in the Midwest to identify any potential geographic patterns. Even though a majority of the patients from their review came from large cities, after accounting for population density, the incidence of firearm-related injuries was higher in smaller/rural cities, with a majority of these being accidental [50]. Along with physical recovery, pediatric patients that survive violent injury have a disproportionate rate of experiencing negative mental and physical outcomes, such as an increased rate of positive PTSD screening and substance use [51]. Survivors of firearm injury and assault constituted another vulnerable population, due to an increased risk of repeat injury [52]. With various factors impacting pediatric gun violence in urban and rural communities, identifying unique ways to decrease primary and repeat injury is vital to quelling this epidemic.

## 5. Head Trauma

Neurological and head trauma occurs within all age groups with many different mechanisms of injury including but not limited to falls, motor vehicle collisions including pedestrian struck and bicycle collisions, violence including assault with and without the use of weapons, penetrating injuries, and sports-related injuries. Head trauma, which is frequently referred to as a “traumatic brain injury” or “TBI”, accounts for approximately 3000 yearly deaths, 29,000 yearly hospitalizations, and 400,000 yearly emergency department visits among children ages 0–14, making this diagnosis the leading cause of disability for children residing in the United States [53]. Incidence and leading injury mechanism vary by age group in children with head trauma [54]. Taylor et al. [54] examined emergency department visits, hospitalizations, and deaths related to TBI from two Healthcare Cost and Utilization Project (HCUP) databases and the National Vital Statistics System (NVSS) and found the rate of TBI related ED visits, hospitalizations, and deaths in 2013 to be 1591.5 per 100,000 for patients aged 0–4, 837.6 per 100,000 for patients aged 5–14, and 1080.7 per 100,000 for patients aged 15–24. The study also found that unintentional falls and being struck by or against objects were the top causes of TBI-related ED visits, hospitalizations, and deaths in patients aged 0–14, while the top causes were falls and motor vehicle crashes in patients aged 15–24 [54]. Within the pediatric population, head trauma frequently occurs due to children having disproportionately larger heads with weak neck muscles, resulting in an increased risk of both cranial injury and intracranial brain matter damage when a traumatic mechanism occurs [55]. These traumatic head injuries have the potential to significantly impact the growth and development of children throughout their lifetime, including performing day to day activities, reaching milestones, and achieving appropriate socialization in order to defeat depression and anxiety.

Within healthcare, many inequities and disparities occur including the recognition and treatment of head trauma in the pediatric population. Within the United States, it has been shown that demographics including race, ethnicity, education level, and socioeconomic status—including income and insurance status—impact the recognition of signs and symptoms of pediatric head injury, the determination of if a patient receives care via pediatrician and/or the emergency department, as well as successful versus detrimental patient outcomes. In relation to race, research reports that black or African American children are less likely to receive medical treatment following head trauma, compared to any other race or ethnicity, with many barriers in place including the opportunity to obtain high-quality healthcare due to a lack in resources such as transportation, the proximity to an academic medical center within urban areas, holding public insurance versus private insurance, and the overall financial burden of obtaining healthcare [56]. In addition to the above-mentioned demographics playing a role in inequities and disparities, it is known that adolescence is the developmental period where risk-taking occurs and is associated with high-risk behaviors and actions such as reckless driving, speeding while driving, driving while under the influence of alcohol and/or drugs, and even violence. Research displays those Black and Hispanic adolescents experiencing head trauma and/or traumatic brain injuries were frequently exposed to violent injuries due to high-risk behaviors such as physical altercations, aggravated assault, gun violence, and homicide corresponding to the exposure of adverse childhood experiences such as growing up in urban neighborhoods with the demographic challenges of finances, transportation, and education [55,57].

The socioeconomic status of families plays a significant role in the inequities and disparities surrounding the healthcare of the pediatric patient with a head injury. It has been estimated that pediatric head trauma costs the nation approximately $667 million annually, with each case costing $1532 and families being liable for roughly 8.3% of all expenditures [56]. This dollar value has the potential to create a financial hardship resulting in both bankruptcy and poor patient outcomes in low income and uninsured households. Research reveals that a family’s income level, health insurance status, and parental educational levels all significantly impact the reporting and treatment of pediatric head injuries. Families classified as having a “high income” estimated 180,335 cases of head trauma, private insurances reported 321,565 cases of head trauma, and parental education level of college or higher reported 318,682 cases of head trauma, whereas lower incomes, public or uninsured, and parental education levels of high school or lower had decreased levels of reporting and treatment [56]. These findings are possibly related to the educational status and yearly incomes in being educated and aware of the symptoms, criteria, and need for treatment with the financial awareness for positive outcomes related to head injuries.

With these multiple barriers in place for minority families, children are not receiving the most appropriate level of care for varied reasons such as education related to injury prevention and treatment, access to healthcare, insurance status, and financial burden, which can result in negative patient outcomes such as permanent neurological damage and deficits as well as death. Due to research proportionate to the inequities and disparities of pediatric head trauma being conducted, the need to aid in the prevention and successful treatment of head injuries is recognized, especially within vulnerable communities.

## 6. Burn Injuries

Pediatric burns exert a lasting effect psychologically, emotionally, physically, and financially on both patients and their families. According to the World Health Organization (WHO), burn injuries are the fifth most common non-fatal injury in children [58]. In the United States, over $211 million dollars were attributed to caring for children with burns [58]. The incidence of burns is higher in children compared to adults, and those below the age of 5 represent the highest risk population [59,60]. Scald and thermal burn injuries are the most common burn injuries seen in children, especially those between the ages of 0–2 [61]. This may be related to the fact that young children are naturally curious and are unable to elucidate the difference between a dangerous and benign object. At that age, they also lack the ability to move away quickly from the causal substance when it comes into contact [61,62]. Compared to older children, infants have thinner skin, which allows for a more severe burn upon exposure to the same substance for the same period of time. This disrupts the integumentary system’s barrier and compromises its protective function, allowing complications like hypothermia, hypotension, and infection to occur [62].

Drago [61] examined 17,237 children aged 5 and under over a six-year period and found that approximately two-thirds of the injuries were related to scald burns and one-third were related to thermal burns. One-year-old children accounted for the largest percentage of each type of burn (38.7% for scalds and 24.8% for thermal burns), and this is thought to be related to the more rapid development of motor skills compared to the cognitive skills required for a child to identify a dangerous object. Scald injuries are frequently seen amongst younger patients due to grabbing a hot object off an elevated surface or spilling a container of hot substance onto them [60,61]. Gender was also found to be a risk factor for burns, with boys composing a larger percentage of affected children in both burn types (58.4% compared to 41.6% females in scald injuries, 54.7% compared to 45.3% females in thermal burns) [61]. Other studies have found a higher rate of thermal contact burns compared to scald injuries [63,64]. When compared to scald injuries, thermal injuries tended to be less severe, affected a smaller area, and usually did not require hospitalization. Thermal injuries also tended to affect the hands and fingers as opposed to the head and upper body in scald injuries in younger patients [61]. This may account for the difference seen in the other studies, as the demographics of those studies are more consistent with an outpatient population. As children grow older, the incidence of scald and thermal contact burns decreases and the incidence of flame burns increases, likely as a result of increasing cognitive development as well as exposure to open flames via cigarettes, open flames in school laboratories, and stovetops [65].

In 2016, there were approximately 8000 pediatric burn admissions, with nearly half occurring in ages 0–4 [66]. Armstrong et al. [66] evaluated the trend of pediatric burn admissions in the United States in children <18 years old between the years of 2003 and 2016. Between 2003 to 2012, there was a decrease in annual admissions to pediatric burn hospitals by 4.6%, and between 2012 to 2016, the rate decreased by 13.4%. Concomitantly, the rate of burn-related emergency room visits also decreased, suggesting that less severe burns are being cared for in the outpatient setting. However, among those that were admitted, Black and Hispanic children made up 47.9% of admitted patients. As these groups tend to historically fall within lower socioeconomic classes, the trend holds that children from lower income families accounted for almost 40% of pediatric burn admissions. A retrospective study at a burn center in Arizona found that the majority of patients in both scald and non-scald burns were Hispanic (63% and 59%, respectively) and that 86% of patient stays were paid for by Medicaid [67]. Lower income families relying on Medicaid tended to have higher complication rates when compared to those with private insurance or self-pay patients. Medicaid patients were also found to have a longer mean length of stay (3.7 days) versus private insurance (3.5 days) and self-pay patients (3.1 days) [68]. Other markers of lower economic status such as payor status, rural vs. urban home address, single vs. binary parenting, prior child protective services reporting, and employment status were related to increased burn injury severity in patients under 16 years old [69,70,71].

Despite the downtrend in pediatric burn admissions over the past couple of decades, the sudden and rapid onset of the COVID-19 pandemic and lockdown brought about an acute change to the daily lives of many people. As a result, the incidence of pediatric burns presenting to emergency rooms during this period increased. D’Asta et al. [72] performed a retrospective comparison of the effects of the lockdown on the pediatric burn population in the UK. They evaluated a five-week period during lockdown and compared it to the same five-week period (April 2020) in the previous year. They noted that despite a decrease in the number of emergency room visits by 60%, the incidence of burn injuries reported was higher, 2.4% compared to 1.5% previously. Scald injuries remained the predominant type of burn injury (85%), but the mean age was found to be 4.8 years compared to 2.9 years, perhaps due to the fact that more children were quarantined at home and may not have had direct supervision at all times. Amongst patients that were admitted, 50% of admissions sustained >5% total body surface area (TBSA) burns with 29% having 10% TBSA burns. In the control year, over 95% of patients presented with a burn less than or equal to 5% TBSA. Amin et al. [73] found that in the early period of the COVID-19 pandemic, the incidence of pediatric head and neck burn injuries increased when compared to the same period during the previous year, again likely related to the inquisitive nature of young children coupled with the inability to directly supervise children for the entirety of the quarantine period as adults were also starting to work from home. Similar findings were documented in retrospective comparisons made in other emergency rooms worldwide [74,75].

As burn injuries are frequently accidental and non-fatal, it remains imperative that clear, bilingual, and accessible education and interventions be implemented to target these populations that are disproportionately affected by burns and their lasting effects on the family unit.

## 7. Orthopedic Trauma

Orthopedic injuries account for the largest proportion of pediatric trauma. The reported rate of fracture, a common pediatric injury, ranges across studies from 12 to 36 per 1000 children annually [76]. Naranje et al. [77] examined the epidemiology of fracture in patients aged 0–19 using the 2010 National Electronic Injury Surveillance System (NEISS) database and US Census information and found that forearm fractures were the most common fracture among pediatric patients, males had a higher risk of fracture than females, and that in patients aged 0 to 14, annual fracture occurrence increased with age [77]. Children aged 10–14 had the greatest risk of fracture when compared to other age groups (0–4 years of age, 5–9 years of age, and 15–19 years of age) [77]. In the US, disparities in orthopedic injury care and outcomes exist within the pediatric population. Malyavko et al. [78] identified pediatric patients with femoral shaft fractures who received open treatment from 2012–2019 through the National Surgical Quality Improvement Program-Pediatric (NSQIP-P) database and evaluated the relationship between race and outcomes following surgery [78]. The study found that 69.1% of patients who had open treatment of femoral shaft fracture were White while 30.9% were from underrepresented minority (URM) groups. In addition, the study found that URM patients were more likely than White patients to have a prolonged length of hospital stay after surgery [78]. Slover et al. [79] conducted a study to evaluate the impact of racial and economic factors on the treatment of pediatric fractures, specifically supracondylar humerus fractures, femoral shaft fractures, and forearm fractures. Study data were obtained from the 2000 KID database. After adjusting for patient sex, age, and region of hospital, Slover et al. [79] determined that percutaneous pinning was used more frequently to treat supracondylar humerus fractures in Black and Hispanic patients than in White patients, and privately insured patients received treatment with an external fixation device for femoral shaft fractures more frequently than Medicaid or self-pay patients. Montgomery et al. [80] also identified disparities in treatment of pediatric supracondylar humerus fractures based on race and economic factors. After evaluating pediatric patients with supracondylar humerus fractures from the 2016 New York Healthcare Cost and Utilization Project’s (HCUP) database, Montgomery et al. [80] determined that non-White patients and patients with lower socioeconomic status were more likely to be treated nonoperatively. Racial disparities exist in diagnostic radiography of fractures in children as well [81]. Baughman et al. [81] evaluated pediatric patients aged 3–18 with upper extremity fractures between 1 April 2017 and 31 July 2021 and found that when compared to White patients, non-White patients with traumatic forearm pain were less likely to receive diagnostic imaging in emergency departments and ambulatory settings and had a decreased likelihood of diagnostic yield in emergency departments. Since orthopedic injury represents a large proportion of pediatric trauma, disparities in orthopedic trauma can significantly impact the pediatric trauma population. Introducing orthopedic screening programs in schools, creating protocols specific to diagnoses, addressing factors influencing disparities, and inquiring with patients about factors prohibiting their access to care can help reduce disparities and prevent injury [82].

## 8. Conclusions

Disparities and inequities exist in pediatric trauma care. This article highlights recent evidence that exist in the topics of access to care, gun violence, child abuse, head trauma, burn injuries, and orthopedic trauma in the United States. Special attention should be paid to addressing these SDOH in our pediatric trauma system. Future directions include initiatives to address inequities before and after injury. Prevention strategies need to be delivered in a culturally competent manner and tailored at the local level. The findings from these studies are important in informing the proper design and environment needed for a more equitable pediatric trauma system.

## Data Availability

Data sharing not applicable.
